# Radiation-induced alteration of apatite on the surface of Mars: first in situ observations with SuperCam Raman onboard Perseverance

**DOI:** 10.1038/s41598-024-61494-5

**Published:** 2024-05-17

**Authors:** E. Clavé, O. Beyssac, S. Bernard, C. Royer, G. Lopez-Reyes, S. Schröder, K. Rammelkamp, O. Forni, A. Fau, A. Cousin, J. A. Manrique, A. Ollila, J. M. Madariaga, J. Aramendia, S. K. Sharma, T. Fornaro, S. Maurice, R. C. Wiens, the SuperCam Science team, Tayro Acosta-Maeda, Christophe Agard, Fernando Alberquilla, Cesar Alvarez Llamas, Ryan Anderson, Daniel Applin, Julene Aramendia, Gorka Arana, Roberta Beal, Pierre Beck, Candice Bedford, Karim Benzerara, Sylvain Bernard, Pernelle Bernardi, Tanguy Bertrand, Olivier Beyssac, Thierry Bloch, Jean-Yves Bonnet, Bruno Bousquet, Abderrahmane Boustelitane, Magali Bouyssou Mann, Matthew Brand, Philippe Cais, Gwenael Caravaca, Kepa Castro Ortiz De Pinedo, Charlene Cazalla, Antoine Charpentier, Baptiste Chide, Elise Clavé, Samuel Clegg, Ed Cloutis, Leire Coloma, Jade Comellas, Stephanie Connell, Agnes Cousin, Lauren DeFlores, Erwin Dehouck, Dot Delapp, Tomas Delgado Perez, Robin Deron, Christophe Donny, Alain Doressoundiram, Gilles Dromart, Ari Essunfeld, Cecile Fabre, Amaury Fau, Woodward Fischer, Hugo Follic, Olivier Forni, Thierry Fouchet, Raymond Francis, Jens Frydenvang, Travis Gabriel, Zachary Gallegos, Cristina García-Florentino, Patrick Gasda, Olivier Gasnault, Erin Gibbons, Martin Gillier, Laura Gomez, Sofia Gonzalez, John Grotzinger, Jennifer Huidobro, Xavier Jacob, Jeffrey Johnson, Hemani Kalucha, Evan Kelly, Elise Knutsen, Gaetan Lacombe, Florentin Lamarque, Nina Lanza, Carene Larmat, Javier Laserna, Jeremie Lasue, Laetitia Le Deit, Stephane Le Mouelic, Chip Legett, Richard Leveille, Eric Lewin, Cynthia Little, Mattéo Loche, Guillermo Lopez Reyes, Ralph Lorenz, Eric Lorigny, Juan Manuel Madariaga, Morten Madsen, Lucia Mandon, Henry Manelski, Nicolas Mangold, Jose Manrique Martinez, Noah Martin, Jesus Martinez Frias, Sylvestre Maurice, Timothy Mcconnochie, Scott McLennan, Noureddine Melikechi, Pierre-Yves Meslin, Frederique Meunier, David Mimoun, Gilles Montagnac, Franck Montmessin, Javier Moros, Valerie Mousset, Naomi Murdoch, Tony Nelson, Ray Newell, Cécile Nicolas, Horton Newsom, Colleen O’Shea, Ann Ollila, Philippe Pantalacci, Jonathan Parmentier, Laurent Peret, Pascal Perrachon, Paolo Pilleri, Cédric Pilorget, Patrick Pinet, Iratxe Poblacion, Francois Poulet, Cathy Quantin Nataf, William Rapin, Ivan Reyes, Laurent Rigaud, Scott Robinson, Ludovic Rochas, Margaret Root, Eloise Ropert, Léa Rouverand, Clement Royer, Fernando Rull Perez, David Said, Pierre Sans-Jofre, Susanne Schroeder, Fabian Seel, Shiv Sharma, Amanda Sheridan, Pablo Sobron Sanchez, Aurélien Stcherbinine, Alex Stott, Michael Toplis, Nathalie Turenne, Marco Veneranda, Dawn Venhaus, Roger Wiens, Uriah Wolf, Allison Zastrow

**Affiliations:** 1 DLR - Institute of Optical Sensor Systems, Berlin, Germany; 2 Institut de Minéralogie, de Physique des Matériaux et de Cosmochimie, CNRS, UMR 7590, Muséum National d’Histoire Naturelle, Sorbonne Université, Paris, France; 3 Earth, Atmospheric, and Planetary Sciences, Purdue University, West Lafayette, IN USA; 4 Laboratoire Atmosphères, Milieux, Observations Spatiales, CNRS, Univ. Saint-Quentin-en-Yvelines, Sorbonne Univ, Guyancourt, France; 5 Research Group ERICA, Universidad de Valladolid, Valladolid, Spain; 6 Institut de Recherche en Astrophysique et Planétologie, CNRS, CNES, Université de Toulouse, Toulouse, France; 7 Los Alamos National Laboratory, Los Alamos, NM USA; 8 Department of Analytical Chemistry, University of the Basque Country UPV/EHU, 48940 Leioa, Spain; 9 Hawaii Institute of Geophysics and Planetology, University of Hawaii, Honolulu, HI 96822 USA; 10 INAF-Astrophysical Observatory of Arcetri, Largo E. Fermi 5, 50125 Firenze, Italy; 11 Centre National d’Etudes Spatiales, Toulouse, France; 12 Universidad de Malaga, Malaga, Spain; 13 U.S. Geological Survey, Flagstaff, AZ USA; 14 University of Winnipeg, Winnipeg, Canada; 15 Institut de Planétologie et Astrophysique de Grenoble, CNRS, Univ. Grenoble Alpes, Grenoble, France; 16 Laboratoire d’Etudes Spatiales et d’Instrumentation en Astrophysique, Observatoire de Paris, CNRS, Sorbonne Univ., Univ. Paris-Diderot, Meudon, France; 17 Institut de Chimie de la Matière Condensée de Bordeaux, CNRS, Univ. Bordeaux, Bordeaux INP, Pessac, France; 18 Université de Bordeaux, Bordeaux, France; 19 Jet Propulsion Laboratory/Caltech, Pasadena, CA USA; 20 ENSL, CNRS, LGL-TPE, Univ. Lyon, Univ. Lyon 1, 69007 Villeurbanne, Lyon, France; 21 GéoRessources, CNRS, Univ. Lorraine, Nancy, France; 22 California Institute of Technology, Pasadena, CA USA; 23 ATOS, Bezons, France; 24 University of Copenhagen, Copenhagen, Denmark; 25 University of New Mexico, Albuquerque, NM USA; 26 McGill University, Montreal, Canada; 27 Institut Supérieur de L’Aéronautique et Espace (ISAE-SUPAERO), Toulouse, France; 28 Institut de Mécanique des Fluides de Toulouse, CNRS, INP, Univ. Toulouse, Toulouse, France; 29 Applied Physics Laboratory, Johns Hopkins University, Laurel, MD USA; 30 Laboratoire de Planétologie et Géodynamique (LPG), CNRS, Nantes, France; 31 Institut Des Sciences de La Terre, CNRS, Univ. Grenoble Alpes, IRD, Univ. Savoie Mont Blanc, Grenoble, France; 32 Planetary Science Institute, Tucson, AZ USA; 33 Agencia Estatal Consejo Superior de Investigaciones Científicas, Madrid, Spain; 34 Space Science Institute, Boulder, CO USA; 35 State University of New York, Stony Brook, NY 11794-2100 USA; 36 University of Massachusetts, Lowell, MA USA; 37 Institut d’Astrophysique Spatiale, CNRS, Univ. Paris-Sud, Orsay, France; 38 Telespazio, Toulouse, France; 39 SETI Institute, Mountain View, CA USA

**Keywords:** Planetary science, Mineralogy, Planetary science

## Abstract

Planetary exploration relies considerably on mineral characterization to advance our understanding of the solar system, the planets and their evolution. Thus, we must understand past and present processes that can alter materials exposed on the surface, affecting space mission data. Here, we analyze the first dataset monitoring the evolution of a known mineral target in situ on the Martian surface, brought there as a SuperCam calibration target onboard the Perseverance rover. We used Raman spectroscopy to monitor the crystalline state of a synthetic apatite sample over the first 950 Martian days (sols) of the Mars2020 mission. We note significant variations in the Raman spectra acquired on this target, specifically a decrease in the relative contribution of the Raman signal to the total signal. These observations are consistent with the results of a UV-irradiation test performed in the laboratory under conditions mimicking ambient Martian conditions. We conclude that the observed evolution reflects an alteration of the material, specifically the creation of electronic defects, due to its exposure to the Martian environment and, in particular, UV irradiation. This ongoing process of alteration of the Martian surface needs to be taken into account for mineralogical space mission data analysis.

## Introduction

A large part of planetary exploration relies on the characterization of minerals throughout the solar system to gain insights into the formation and evolution of planets from the identified mineral assemblages, compositions, textures, etc. Several techniques have been used to achieve these objectives. Among them, the use of Raman spectroscopy for planetary exploration has been investigated for decades, highlighting the interest of this technique for mineralogical and organic analysis^1–12^. Several Raman instruments have been proposed and developed for in situ exploration missions, particularly for Mars^13–16^ and for one of its moons^17,18^. Since February 18th, 2021, NASA’s Perseverance rover has used a suite of scientific instruments to explore the Jezero Crater, Mars^19,20^. Part of this payload is the SuperCam instrument, which, together with the SHERLOC instrument^14^, enables the use of Raman spectroscopy for the first time for in situ planetary exploration^15,16^. These two Raman instruments have contributed significantly to addressing the scientific objectives of the Mars 2020 mission by enabling direct detection and characterization of mineral phases, including perchlorate, sulfate, olivine and carbonate, in Martian geologic targets^21–28^. The SuperCam instrument also enables chemical analyses with laser-induced breakdown spectroscopy (LIBS), mineralogical analyses with visible and near-infrared reflectance spectroscopy (VISIR), texture characterization with a remote microimager (RMI) and acoustic measurements with a microphone^15,16^.

To better understand the data acquired with these instruments—and in situ sensing in general—and to accurately interpret the implications for mineral presence and assemblages, one needs to understand whether and how Martian environments may affect minerals. In particular, in the absence of a thick atmosphere, the surface of Mars receives a greater flux of solar electromagnetic radiation than does Earth. This extra amount of radiation concerns the whole spectral range of solar radiation, and in particular, ultraviolet (UV) photons in the range of 190–410 nm reach the Martian surface without attenuation. Moreover, in the absence of a global magnetic field, the ionizing radiation of solar energetic protons and galactic cosmic rays^29–31^ also reach the Martian surface. High-energy radiation can penetrate the ground and affect materials to a depth of approximately two meters, whereas UV radiation penetrates only a few millimeters at most.

Relatively few studies have been dedicated to the effects of irradiation on minerals in the Mars environment, and the influence of billions of years of exposure of Martian rocks to this environment is not well understood. Several studies have shown that irradiation (ions, gamma-rays, etc.) of inorganic samples (e.g., quartz, SiC) results in modification of the structure of the sample and induces different kinds of electronic defects^32–34^. These radiation-induced modifications are evidenced by a reduced Raman signal, broadening and, in some cases, shift of the Raman bands^34^, and an increased continuum signal. Particularly applicable to Martian exploration, a recent study showed that UV irradiation may significantly alter the spectroscopic signature of hydrated minerals and generate electronic defects in different minerals^35^. These defects are responsible for a strong increase in the intensity of the continuum signal in the Raman spectra, as well as a decrease in the Raman signal-to-background ratio. These studies raise the possibility that minerals on the surface of Mars might be altered through prolonged exposure to UV radiation. This could significantly affect the detection of minerals on Mars via vibrational spectroscopy techniques such as Raman or infrared reflectance spectroscopy. It is therefore crucial to investigate the influence of UV radiation on minerals at the surface of Mars to further improve mineral detection and characterization.

For this study, we used the SuperCam Raman instrument to monitor the evolution of one of SuperCam’s dedicated onboard calibration targets (SCCTs^36^), an apatite (TAPAG), throughout the first 950 Martian days (sols) of the mission. This target was chosen for its relatively strong Raman signal on Mars, and for the scientific interest of phosphate mineral characterization in the context of planetary exploration^37^. We analyze the Raman spectra acquired on Mars and their evolution during the mission thus far. To support the interpretation of this unique dataset, we compare it to spectra acquired in the laboratory, where a similar apatite target was irradiated with a UV lamp under low pressure and temperature conditions, to simulate the Martian UV radiative environment. We discuss the alteration of an apatite target in the real Martian environment, the representativeness of laboratory UV-radiation experiments, and the implications for mineralogical analyses of rocks at the surface of Mars. A detailed description of the instruments and the data processing methods are presented in the “Methods” section at the end of the manuscript. The observations are reported and discussed in the following sections.

## Results

### Mars data

SuperCam uses a green, pulsed laser to excite Raman signal in targets located up to 10 m from the instrument^15,16^. The scattered light is collected with a telescope, injected into an optical fiber and then into a transmission spectrometer equipped with an intensified CCD. Light is collected during a 100 ns window around the laser pulse to minimize daylight and luminescence inputs. The design, characteristics and performances of the SuperCam instrument, as characterized prelaunch, have been described in detail in previous publications^15,16^. In this study, each Raman spectrum is normalized to the mathematical mean of the signal across the 240–2000 cm^−1^ spectral range to account for signal variability due to temperature variations in the instrument between measurements.

**Figure 1:**
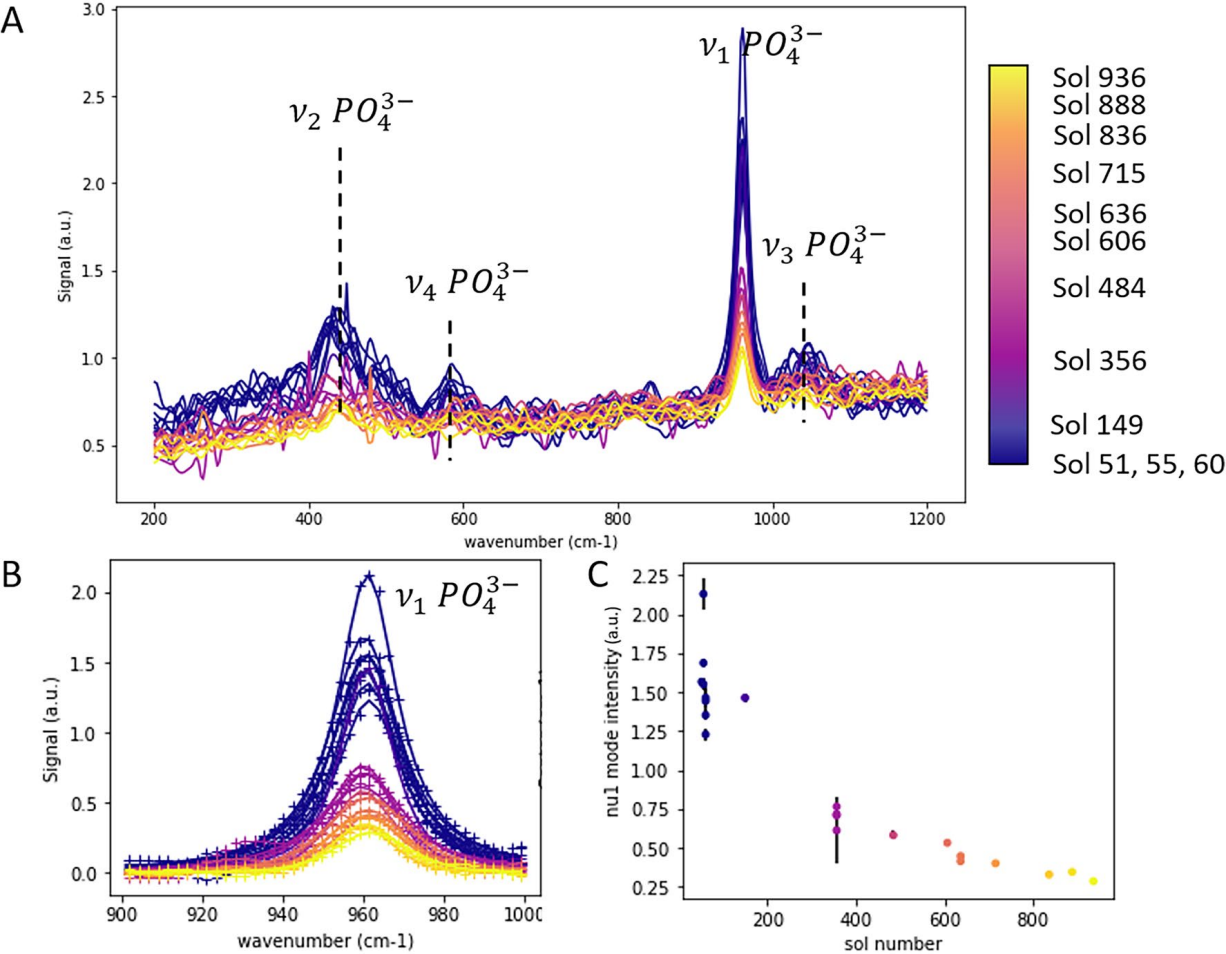
Mars Raman spectra acquired with SuperCam on apatite SCCT (TAPAG) on different sols throughout the mission, normalized to the mean signal. The plots are color coded with sol number. (**A**) Normalized spectra (x axis shows wavenumber in cm^−1^); (**B**) Close-view in the 900–1000 cm^−1^ spectral range; baseline-corrected experimental data ( +) and fitted pseudo-Voigt profile (full lines). (**C**) Intensity of the $\nu_{1}$ mode of apatite extracted from the normalized spectra (area of the fitted pseudo-Voigt profile) as a function of the sol of the observation. The error bars represent the uncertainty values of the parameters derived from the peak fitting procedure.

We used Raman spectra acquired on the TAPAG target on twelve different sols between sols 51 and 928 (details in Table S1). The normalized spectra are presented in Fig. 1A (nonnormalized data in Figure S1). The most significant feature is the ${\nu }_{1}$ mode of apatite (symmetric stretching of PO_4_^3−^), observed at approximately 960 cm^-1^ in all the spectra (Fig. 1B). In the data acquired at the beginning of the mission, additional modes of apatite are also detected, mainly the ${\nu }_{2}$ (bending of PO_4_^3−^) and ${\nu }_{3}$ (triply degenerate antisymmetric stretching of PO_4_^3−^) modes, approximately 440 cm^−1^ and 1055 cm^−1^, respectively, and possibly also the ${\nu }_{4}$ (triply degenerate bending of PO_4_^3−^) mode, approximately 600 cm^−1^ (Fig. 1A). These weaker modes are no longer visible after a few hundred sols on Mars. For this reason, we focus on the ${\nu }_{1}$ mode and track its evolution.

As the target spends more time on Mars, we observe a clear decrease in the relative contribution of the Raman signal to the total signal (factor 8 difference in normalized peak area, Fig. 1C) due to both an increase in the continuum signal and a decrease in the Raman signal (Figure S1). By monitoring the other characteristics of the Raman signal, such as peak position or width, we did not observe any other significant trend with increasing sol number (Figure S1).

### Laboratory irradiation experiment

In the laboratory, we simulated Martian conditions by placing a replicate of the TAPAG SCCT in a Martian chamber under low pressure (~ 1 mbar) and temperature (< 0 °C) and exposing it to UV irradiation. The spectrum of the lamp is similar to the solar light received on the Martian surface (details in Method section). With a SuperCam-like Raman system^35,38,39^, we acquired Raman spectra in situ in the chamber at different times over an ~ 24 h-long continuous irradiation period.

**Figure 2:**
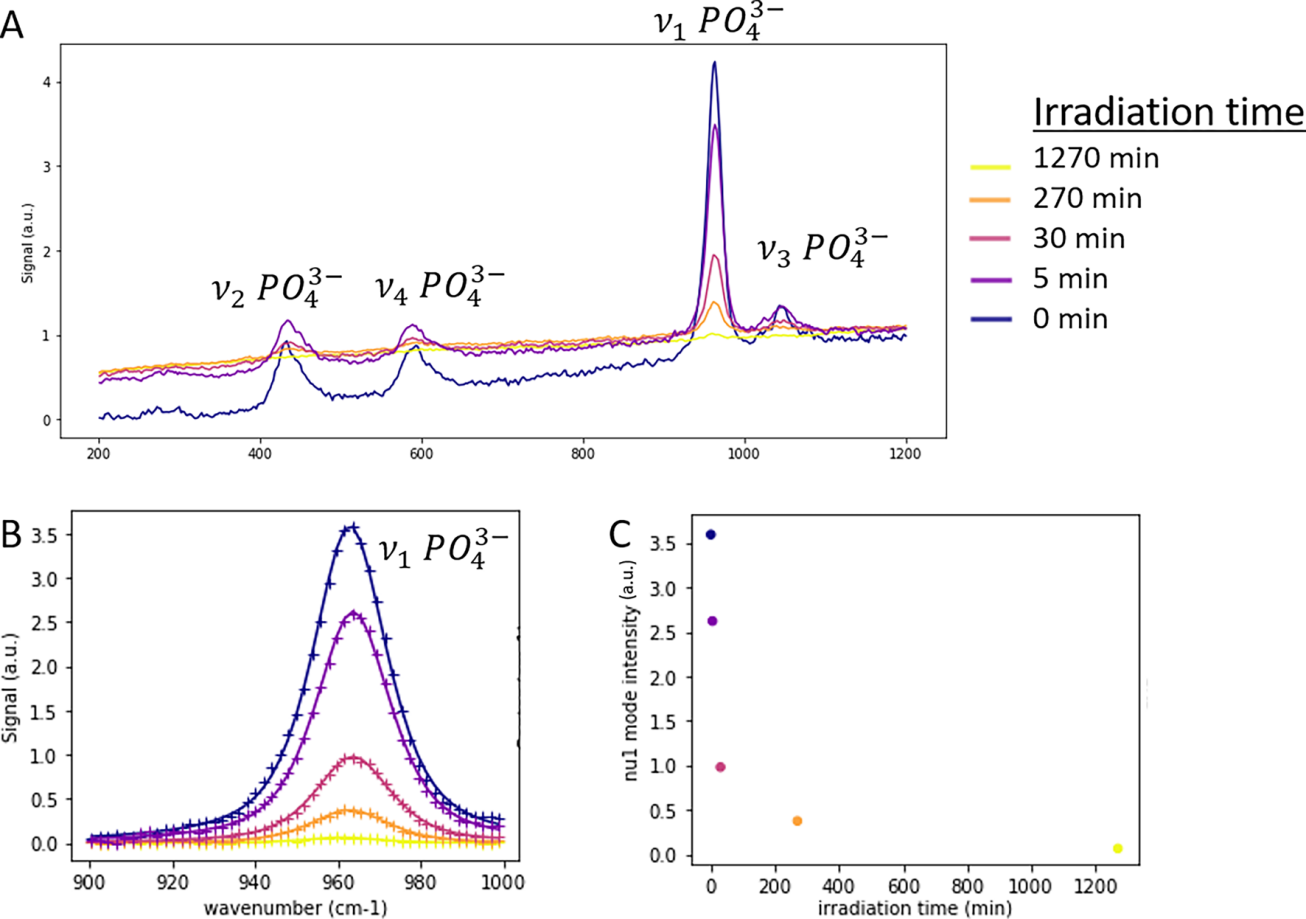
Laboratory Raman spectra acquired on an apatite target after different durations of UV irradiation (0 min, 5 min, 30 min, 270 min, 1270 min), normalized to the mean signal. The plots are color coded with irradiation time. (**A**) Normalized spectra (x-axis shows wavenumber in cm^−1^); (**B**) Close-view in the 900–1000 cm^−1^ spectral range; baseline-corrected experimental data ( +) and fitted pseudo-Voigt profile (full lines). (**C**) Intensity of the $\nu_{1}$ mode of apatite extracted from the normalized spectra (amplitude of the fitted pseudo-Voigt profile) as a function of the duration of target irradiation. The error bars represent the uncertainty values of the parameters derived from the peak fitting procedure (smaller than the markers, in this case).

The spectra acquired in the laboratory at different irradiation durations on the apatite target are presented in Fig. 2. We observed good-quality Raman spectra, with several Raman modes of apatite visible in each spectrum (Fig. 2A). Overall, the contribution of the Raman signal to the spectra decreases with increasing irradiation time (Fig. 2B,C). Other characteristics of the Raman ${\nu }_{1}$ mode are presented in Fig. S2. The width of the peak appears mostly stable (within error bars) throughout the series.

## Discussion

### Possible sources of evolution of the Raman signature of the apatite calibration target

We observe a clear and significant evolution of the Raman spectra acquired on the apatite SCCT throughout the first 950 sols of the Mars 2020 mission, with a decreasing contribution of the Raman apatite signal to the total signal.

#### Evolution of the instrument?

Two other calibration targets, namely, a diamond and white paint, are used to monitor the health and eventual aging of the instrument^36^ (section “The target”). As with the apatite SCCT, Raman spectra were acquired regularly on these two targets throughout the mission, and they do not show trends similar to those observed for apatite (example shown in Fig. S3). This suggests that the observed evolution of apatite is not a result of changes in the instrument response but rather an effective evolution of the apatite target.

Even though certain environmental or acquisition parameters appear to affect the Raman spectra (e.g. the surface temperature at the time of analysis which affects the electronics as discussed in Bernard et al., submitted), which results in some remaining variability in the data even after normalization, the time spent on the surface of Mars appears to be the most significant parameter in the evolution of apatite spectra.

#### Dust layer or mechanical alteration of the target?

As the mission progresses, the surface of the apatite SCCT is altered in two ways that may affect the Raman spectra.

**Figure 3:**
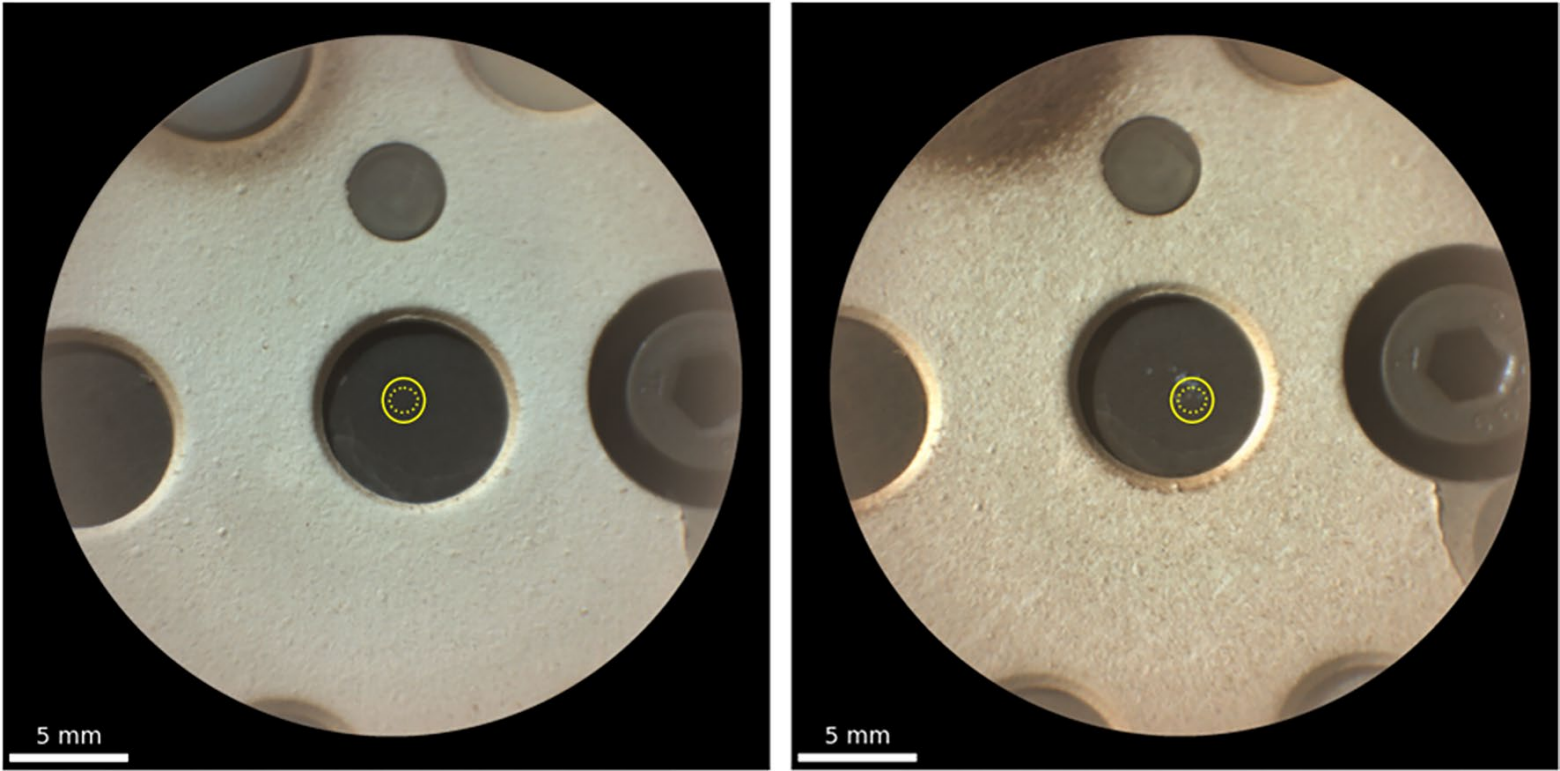
Remote microimages (RMIs) of the apatite SCCT acquired on Mars with SuperCam, corresponding to the first and one of the latest Raman spectra considered in this study (sols 51 and 836). The yellow ellipses represent the fields of view of the Raman observation (dashed: 68%; full line: 95%).

First, as shown in Fig. 3, dust may be deposited on the surface of the calibration targets, which could affect the Raman spectra. In particular, it was shown in previous studies that a layer of dust on top of a crystal could result in (i) a decreased Raman signal from the crystal or (ii) an increased continuum signal^16,40^. However, in this case, we have strong arguments to think that this effect is not significant. First, the target holder is tilted (~ 50 degrees from the rover deck) to reduce the amount of dust deposited on the targets^16,41^. Moreover, most SCCTs, including apatite, are regularly analyzed via LIBS (approximately every 100 sols), and the shockwaves resulting from LIBS analyses are known to blow dust from the surface of the target, on an area of several millimeters around the LIBS point^5,15,42^. It is therefore unlikely that a significant layer of dust will accumulate on these targets. Finally, a specific test was performed on Mars to observe the influence of dust on the Raman spectra acquired with SuperCam. For three different targets, including the TAPAG, spectra were acquired on the same spot before and after LIBS analysis. Looking at the LIBS data, we see the contribution of dust in the spectra corresponding to the first two laser shots and not in the following spectra, indicating that some dust was indeed blown away by the LIBS observation. The Raman spectra do not show significant, repeatable differences between the before- and after-LIBS observations, which would correspond to the contribution of dust to the Raman spectra. The two spectra acquired on the apatite SCCT for this test were included in this study (sol 636, Table S1).

Note that it is possible that a small, electrostatic portion of dust might stick to the target and not be blown away by the ablation-related shockwave. However, this would have to be a small portion of the dust, as we don’t see it in post-LIBS RMI images. Additionally, the contribution of this remaining dust to the Raman signal should be similar to (or weaker than) the contribution of the dust that is blown away, which we showed to be negligible.

Additionally, every LIBS analysis performed on this target creates an ablation crater, modifying the surface of the target as the mission progresses (Fig. 3). Several studies have been dedicated to determining the influence of LIBS analysis on subsequent Raman observations^38,43,44^. All of these methods highlight potential modifications of the targets due to LIBS analysis within but also close to the ablation crater, such as amorphization, melting and/or phase transition. Additionally, interactions with the atmosphere may result in the formation of oxides or the deposition of carbonaceous material^43^. Fau et al.^38^ showed that the modified area was sufficiently smaller than the SuperCam Raman field of view so that it did not significantly impact the Raman observations performed after LIBS with SuperCam (diameter of LIBS spot ~ 200 μm vs ~ 1.3 mm Raman footprint). However, this approach is used for a single LIBS crater within the Raman field of view. In the case of the apatite target in this study, multiple ablation craters—and surrounding altered areas—may be present within the field of view. Moreover, it is not clear how this specific material—sintered synthetic apatite—may be affected by laser ablation. To characterize the possible influence of multiple LIBS craters on the Raman spectra acquired on the apatite SCCT, we performed a complementary test in the laboratory using a copy of the apatite SCCT onboard Perseverance.

At DLR in Berlin, Germany, we created a laser ablation crater corresponding to 10 laser shots under Martian-like atmospheric conditions using the LIBS setup described in previous studies^43^ and a laser pulse energy of ~ 10.5 mJ. These conditions are comparable to the LIBS observations of SCCTs on Mars^25^. We then analyzed pristine and LIBS-affected areas with micro-Raman using a setup described in previous studies^43^. To characterize the influence of previous laser ablation methods on the Raman spectra, the Raman and continuum signal intensities were extracted in the same way as for the other datasets presented in this study. The test and results are shown in Fig. S4. We note that multiple Raman modes of apatite were detectable in every single spectrum acquired on the target. Despite the significant variability in the Raman signal, as well as in both the continuum shape and intensity, we observed no significant difference between the Raman and continuum signals recorded in LIBS-affected and pristine areas. To conclude, there is no evidence that the Raman spectra acquired with SuperCam on Mars should be affected by LIBS-induced craters.

Beside these surface effects, the only thing that could alter the target enough to change its Raman spectrum is the Martian environment. Based on the literature, as well as the laboratory data presented in this study, we discuss whether the changes observed in the Raman spectra of the SuperCam apatite calibration target are consistent with radiation-induced alteration.

### Radiation-induced aging on Mars?

In the literature, the majority of studies on radiation-induced alteration of materials are dedicated to organic materials. Indeed, since the Viking and Phoenix landers (1976 and 2008, respectively) reported the absence of any detectable organic compound on the surface of Mars^45^, even though it is expected that micrometeorites and space dust should bring a constant flux of organic matter to the surface^46,47^, it has been hypothesized that the radiative environment at the surface of Mars may alter organic compounds. Consequently, a large number of studies have been dedicated to determining the influence of the Martian radiative environment on organics by simulating the Martian radiative environment in the laboratory and observing the evolution of different organic compounds when subjected to this environment^39,48–50^. Additionally, the influence of different minerals mixed with organic compounds has also been investigated^51–54^. These studies mostly rely on both infrared and Raman spectroscopy techniques, as both enable characterization of the structure and molecular bonds and are sensitive to some electronic defects in both organic and inorganic samples. Overall, these studies have shown that UV irradiation at the surface of Mars may significantly alter organic compounds within a few days to a few months^48,49^. Yellowing of the samples was observed, indicating the creation of color centers^39^. This can be accompanied by a decrease in the sample’s reflectance and/or an increase in the continuum signal in the Raman spectra^39^. The characteristic absorption bands in infrared reflectance spectroscopy, as well as Raman modes, show decreasing intensity with increased irradiation time^49,52^, highlighting the alteration of the compounds, either through fragmentation or polymerization^49^. Moreover, the appearance of new spectral features sometimes reveals the formation of new molecular bonds in photoproducts^48,54^. Notably, the interaction of organic compounds with minerals may significantly modify the behavior of organic compounds under UV irradiation, sometimes increasing the decay of the molecule and sometimes shielding it from UV degradation.

Note that the first studies on the aging of organic targets in situ in the actual Martian environment are ongoing using the SuperCam and SHERLOC instruments and their calibration targets^36,55,56^.

Compared to the extensive literature on organics, relatively fewer studies have been dedicated to the influence of irradiation on minerals of the type experienced on Mars. A number of studies have investigated radiation from radioactive minerals in close proximity^57–62^. It has been shown that irradiating minerals can result in a decreased contribution of the Raman signal to the total signal, and this observation is explained by the creation of color centers and/or electronic defects in the sample^35^. It is proposed that the increased background could reflect luminescence from these defects. Additionally, these defects can increase the opacity of the target, resulting in a reduced laser-excited and Raman-probed volume and hence a reduced Raman signal.

In both the Mars experiments and UV irradiation in the laboratory, we observed a decrease in the relative contribution of the Raman signal to the total signal. This is likely due to the creation of electronic defects in the sample by irradiation. As mentioned for organic-bearing targets, these electronic defects act as luminescence centers, inducing an increased continuum, but also result in increased absorption of visible light by the target and hence in a reduced probed volume in the target, leading to a decreased contribution of the Raman signal to the total signal.

This evolution of the spectra is not accompanied by significant changes in the Raman signal (position, width) on Mars, indicating that phase transformation or amorphization are unlikely. Some variability in the position of the ${\nu }_{1}$ mode of apatite is observed in the laboratory series (Fig. S2), but these variations are not accompanied by a broadening of the mode and no notable trend is observed in the other Raman modes of apatite visible in the spectra (Fig. S5). This variability is thus attributed to instabilities in the system and not considered to be significant.

The durations of exposure monitored on Mars (almost 1000 sols) and in the laboratory (1270 min) are very different, making it difficult to compare the kinetics of the observed process. Considering that the spectrum of UV-irradiation in the laboratory is representative of that of solar irradiation on the surface of Mars^35^ but that illumination in the laboratory was constant and continuous for the whole length of the experiment without shadowing or a day-night cycle, as experienced by the target at the surface of Mars, we estimate that the dose received by the sample in the laboratory is consistent with the dose received by the SCCT on Mars over a few days to one week^35^. Note that the impact of a continuous irradiation of the target compared to the interrupted irradiation related to the day-night cycle on Mars has not been documented; this topic should be studied in the future. Additionally, observations of the apatite target on Mars started only on sol 51 due to operational constraints. If the radiative environment on Mars is actually altering the target, it would have started affecting it from sol 1. Therefore, we cannot observe the exact same regimes in the laboratory or on Mars, as the laboratory data record the effect of the first exposure to UV radiation on the target.

Overall, the evolution of the Raman spectra observed on Mars and under UV irradiation in the laboratory are consistent, indicating that (i) the evolution of the apatite target observed with Raman spectroscopy on Mars is likely due to UV radiation-induced alteration; (ii) vibrational spectroscopy data acquired on Martian rocks should be interpreted carefully, as the minerals may have been affected by radiation; and (iii) UV irradiation tests in the laboratory are relevant for simulating the Martian radiative environment and studying its influence on samples. However, further studies are necessary to understand how alteration kinetics can be compared between laboratory and in situ experiments, taking into account possible relaxation periods due to shadowing or day-night cycles, as well as the influence of other parameters such as pressure and temperature.

This may have significant consequences for the use of Raman spectroscopy for geologic investigations, particularly in the context of space exploration. It has been understood for some time now that the search for organic molecules and biosignatures on Mars should be focused on subsurface material, where organic molecules might be shielded from destructive radiation and thus preserved and detectable. This study showed that similar precautions might be appropriate for analyses of some minerals as well. In particular, certain minerals exposed to UV irradiation may be challenging to characterize with Raman spectroscopy. This study only looked into the influence of UV irradiation on an apatite mineral. Laboratory experiments performed by Royer et al. showed that Raman signatures of different phosphates, sulfates and carbonates also change with the duration of Mars-like UV irradiation^35^. It is therefore likely that other minerals are similarly affected by UV radiation on the Martian surface as the apatite. This raises significant questions regarding the use of Raman spectroscopy for space exploration. Despite these difficulties, both the SuperCam and SHERLOC Raman instruments are able to detect several mineral signatures, including olivine, sulfate, carbonate, phosphate and perchlorate, in a significant number of geologic targets on the surface of Mars^20–27^. A significant portion, though not all, of these detections were made on abraded surfaces obtained by mechanical abrasion of the first 5 to 10 mm of a rock surface. Abrading thus appears to be a relatively easy way to circumvent the UV radiation-induced alteration of samples on the surface of Mars. Further studies will be needed to fully understand this UV-induced alteration on different minerals and the full extent of the implications for space exploration.

## Conclusion

We present the first in situ observations of the evolution of a mineral target exposed to the Martian environment, as observed with the SuperCam Raman instrument. The Raman spectra obtained from the SuperCam apatite calibration target over the first 950 Martian days of the Mars 2020 mission present a decreasing contribution of the Raman signal to the total signal. Overall, this evolution is consistent with the changes observed when irradiating a similar target with UV photons in the laboratory to simulate the Martian environment. To conclude, mineral targets can be altered by the environment on Mars within a matter of weeks (if not faster than that). This should be taken into account when interpreting Raman data and possibly data resulting from other vibrational techniques acquired in situ by space exploration probes.

## Materials and methods

### Mars data

#### SuperCam Raman instrument: architecture, data acquisition and processing

The design, characteristics and performances of the SuperCam instrument, as characterized prelaunch, are precisely described in previous publications^15,16^. Briefly, with a frequency-doubled Nd:YAG laser, 4 ns, 532 nm laser pulses are fired at targets located 1.5 to 10 m from the instrument. For Raman spectroscopy, a repetition rate of 10 Hz is used with 9 mJ per pulse deposited on an ~ 1 cm-diameter spot. The signal resulting from the interaction between the laser pulse and the target is collected with a Schmidt-Cassegrain telescope in the Mast Unit^15^. A Notch filter rejects 96% of photons in the 531–533 nm range, while the remaining light is coupled into a 6 m-long optical fiber to the Body Unit^16^. There, it is guided through a series of dichroic mirrors to split the light into the corresponding spectrometers. Finally, an edge filter cuts off the laser light (under 534 nm) before guiding the photons in the range of 532.0 to 858.8 nm in a transmission spectrometer equipped with an intensified CCD camera. The optical arrangement provides a 0.7 mrad (e.g., ~ 1.1 mm diameter footprint at 1.5 m) field of view on the surface of the analyzed target, which is coaligned within the area illuminated by the laser on the target.

The gated CCD and Q-switched laser provide temporal resolution capabilities, enabling SuperCam to acquire signals at intervals as short as a 100 ns window adjusted to catch the arrival of backscattered light from the laser pulse. This gated acquisition reduces the noise coming from both ambient light and possible luminescence in the sample^10,37^. This approach also allows the amplification of signals with different gain values. The signal corresponding to several laser shots can accumulate on the CCD; the resulting spectrum is called a coaddition *(co-add)*. For each *co-add*, one dark spectrum is acquired with the same parameters as the active spectrum but without the laser. This dark spectrum is then subtracted from the active spectrum to remove ambient light and electronic noise. Several *co-adds* are generally acquired, and the spectra are then averaged to maximize the signal-to-noise ratio of the features present in the spectra. The Raman spectra acquired with SuperCam on Mars generally result from 100, 200 or 400 laser shots per point, distributed in varying combinations of *co-adds*.

In this study, we used SuperCam-calibrated spectra, available from the PDS^63^. The preprocessing of Raman spectra includes the following steps: spike removal through a sigma-clipping algorithm, dark subtraction as mentioned before, stitching together adjacent spectral ranges, denoising using a wavelet transform, wavenumber calibration, and instrument response function (IRF) correction^64^.

#### The target

The SuperCam instrument includes a series of carefully assembled calibration targets (SCCTs), which are located at the backside of the rover and are regularly analyzed with different techniques^36,41^. Two calibration targets are specifically designed for the Raman instrument: a diamond and an organic target. The diamond, being a stable material and thus expected to remain unaltered upon exposure to the Martian environment, is used for regularly checking instrument health based on peak position (wavelength calibration) and peak intensity (instrument health, mostly alignment), as described previously^36,41^. The organic target is an ertalyte used in case studies to monitor the aging of organic compounds in the Martian environment^55^. A third target is regularly used for Raman calibration: the white paint of the SuperCam calibration target holder; this paint was specifically developed to withstand the harsh Martian conditions. In this study, we use the Raman data obtained from another SuperCam calibration target, apatite (TAPAG), which was designed for LIBS calibration but provides a good Raman spectrum with multiple modes. Note that other mineral targets among the SCCTs would have been targets of interest for this study, but the LIBS-oriented preparation of the targets, including crushing to fine grains and spark plasma sintering^36,65^, preserved the chemical composition but in some cases altered the mineralogy of the targets^66^. As a result, some of these targets do not have a detectable Raman signal on Mars (e.g. the olivine target) and some others require further work to fully understand their Raman spectra (e.g. the calcite target). The TAPAG target is therefore the mineral target with the strongest Raman signal among the SuperCam calibration targets, which made it ideal for this study.

Apatite is a calcium phosphate with the theoretical formula (OH, Cl, F)Ca_5_(PO_4_)_3_ (hydroxyl, chloro- and fluoroapatites, respectively). It is a common mineral in terrestrial rocks and meteorites and was previously identified on Mars^67,68^ and is ubiquitous in Martian meteorites^69,70^. Apatites can be magmatic, sedimentary or biological and can trace fluid‒rock interactions, giving them particular interest in the context of planetary exploration^37^. Apatite is easily characterized via Raman spectroscopy^37,71^, and the different Raman modes of apatite are extensively characterized in the literature^71^, with the different phosphate modes usually found at the following positions: ${\nu }_{2}$ at approximately 440 cm^−1^, ${\nu }_{4}$ at approximately 600 cm^−1^, ${\nu }_{1}$ at approximately 965 cm^−1^, and ${\nu }_{3}$ at approximately 1055 cm^−1^. In hydroxyapatites, additional modes can be observed related to the OH group: OH translation at 337 cm^−1^, OH^−^ libration at 655 cm^−1^ and OH^−^ stretching at 3573 cm^−1^.

As described by Cousin et al.^36^, TAPAG SCCT is a synthetic sample that was “designed stoichiometrically, and its composition was checked frequently using the EMPA technique, which demonstrated the good homogeneity of the sample within RSDs < 12%”. It contains 54.85 wt% CaO (0.37% RSD), 41.61 wt% P_2_O_5_, 2.98 wt% Cl, 1.82 wt.% F and 0.016 wt% H. The powdered apatite was sintered using spark plasma sintering^65^ under an argon atmosphere, resulting in a ceramic-like pellet^36^.

#### Observations of the apatite calibration target on Mars

The apatite calibration target offers one of the best Raman signals of the mineral targets among the SCCTs; hence, the SuperCam team has employed this target in the calibration effort of the SuperCam Raman instrument. Consequently, we have acquired a significant number of Raman spectra since the beginning of the mission (between April 2021 and November 2023), although some of these spectra were acquired under different experimental conditions. In this study, we used 21 Raman spectra acquired on the TAPAG target on 12 different sols between sols 51 and 928 (details in Table S1).

In the Raman spectra acquired on the SCCTs, we observe variability in the signal (both Raman and background) with variations in the instrument temperature. This variability is efficiently corrected by normalizing the spectra to the total signal (see Figure S3 and Bernard et al., submitted). We therefore normalize our spectra to the mean signal (equivalent to the total signal, but gives larger intensity values, which is easier to read).

#### Processing the Raman spectra and extracting the characteristics of key features

To characterize the effect of UV irradiation on the Raman spectra acquired on the apatite, in addition to an overall comparison of the spectra, we extracted the intensity of the signal of interest. To do so, we fit the main Raman mode of apatite with a pseudo-Voigt profile, which effectively models the convolution of the Lorentzian profile expected for Raman modes in theory, with the Gaussian contribution of the instrument^72^. We use the nonlinear least-square minimization and curve fitting Python library (LMFIT)^73^ and fit the baseline locally around the peak with a linear function. From the fitted mode, we use the peak area to monitor the signal intensity; we also track the line width ($\sigma$) and position of the center of the line. We use the uncertainty of these parameters, as provided by LMFIT, to characterize the error in the fits.

To compute the noise, we fit the baseline of the spectra in the 1300–1600 cm^-1^ spectral range, which does not contain Raman modes for apatite with asymmetric least squares (AsLS) smoothing^74^; the value of the noise is the standard deviation of the signal in that range after baseline subtraction. To compute the signal-to-noise ratio (SNR), we divided the height of the fitted peak by this noise value.

### Laboratory tests and data

The SuperCam-like remote Raman test bed used for this study has been described in several previous studies^38,39^, and the test protocol used in this study is similar to that described in Royer et al.^35^. The main characteristics of the setup are summarized here.

A laser is collimated at the surface of the sample, located 8 m away from a commercial 200 mm Schmidt Cassegrain telescope, to reproduce SuperCam’s long-distance observation conditions. The 532 nm laser generates pulses of 1.2 ns at a repetition rate up to 2 kHz. An irradiance of 10^10^ W/m^2^ is deposited on an ~ 6-mm diameter spot on the surface of the sample, corresponding to a sufficiently low laser irradiance to prevent any laser-induced damage on the sample^38^. The Raman signal is collected by a telescope with a field-of-view of ~ 5 mm diameter and aligned with the laser; the collected signal then goes through an edge and a notch filter to cut off Rayleigh scattering (below 90 cm^−1^), is injected into an optical fiber, and hence into a modified Czerny-Turner spectrometer (Princeton IsoPlan 320) coupled with an intensified CCD camera (iCCD, Princeton Instruments PIMAX4). A 600 line/mm grating is used within the spectrometer, yielding a spectral resolution of 10–12 cm^−1^, which is similar to SuperCam’s resolution. To maximize the signal-to-noise ratio of the laboratory data, spectra were acquired with a 2 ns gate centered on the laser pulse, and one million shots are accumulated for each spectrum, with a repetition rate of 2 kHz. The difference in gate width with Mars data (2 ns vs 100 ns) is expected to result in different Raman-to-background ratios between the two series, but this does not affect our interpretations, as only relative evolutions are used here and the Mars and laboratory data are not directly compared to each other.

The sample was placed in a Martian simulation chamber, which was described in previous studies^35,39^. The sample was exposed to UV radiation at low temperature (< 0 °C) via a Peltier device in the sample holder under a primary vacuum (~ 1 mbar). The pressure and temperature conditions were set 15 h before starting irradiation to ensure that the observed evolution of the sample was not due to degassing in vacuum. UV radiation is produced by a 150 W arc lamp equipped with a high-pressure xenon bulb (UXL-150SP-LOT-ORIEL). The resulting UV irradiation (190–400 nm) has a spectrum similar to that of solar radiation at the Martian surface, as well as a flux on the same order, providing a good approximation of Martian conditions, as detailed in Royer et al.^35^. The lamp is equipped with a DUV Grade Fused Silica window, enabling rejection of most of the IR radiation (down to 3 $\mathrm{\mu m}$) and thus minimizing heating of the sample by the UV lamp. Raman spectra were acquired in situ in the chamber before turning the UV lamp on and after 5 min, 30 min, 270 min and 1270 min of irradiation, thus enabling monitoring of the samples under UV radiation. Background spectra are acquired by shutting the photocathode to characterize the noise on the detector. These background spectra are subtracted from the spectra.

These laboratory spectra were processed in the same way as the Mars data, including normalization and fitting of the apatite ${\nu }_{1}$ mode (described in section “Processing the Raman spectra and extracting the characteristics of key features”).

### Supplementary Information

Supplementary Information.

## Data Availability

SuperCam data are made available on the PDS. Laboratory data will be made available upon request addressed to the corresponding author (Elise Clavé, elise.clave@dlr.de).

## References

[1] Wang A, Haskin LA, Cortez E (1998). Prototype Raman spectroscopic sensor for in situ mineral characterization on planetary surfaces. Appl. Spectrosc..

[2] Sharma SK, Angel SM, Ghosh M, Hubble HW, Lucey PG (2002). Remote pulsed laser Raman spectroscopy system for mineral analysis on planetary surfaces to 66 meters. Appl. Spectrosc..

[3] Popp J, Schmidt M (2004). Raman spectroscopy breaking terrestrial barriers!. J. Raman Spectrosc..

[4] Wiens RC, Sharma SK, Thompson J, Misra A, Lucey PG (2005). Joint analyses by laser-induced breakdown spectroscopy (LIBS) and Raman spectroscopy at stand-off distances. Spectrochim. Acta Part A Mol. Biomol. Spectrosc..

[5] Sharma SK, Misra AK, Lucey PG, Wiens RC, Clegg SM (2007). Combined remote LIBS and Raman spectroscopy at 8.6m of sulfur-containing minerals, and minerals coated with hematite or covered with basaltic dust. Spectrochim. Acta Part A: Mol. Biomol. Spectrosc..

[6] Tarcea N, Frosch T, Rösch P, Hilchenbach M, Stuffler T, Hofer S, Thiele H, Hochleitner R, Popp J, Botta Oliver, Bada Jeffrey L, Gomez-Elvira Javier, Javaux Emmanuelle, Selsis Franck, Summons Roger (2008). Raman spectroscopy—A powerful tool for in situ planetary science. Strategies of life detection.

[7] Sharma SK, Misra AK, Lucey PG, Lentz RCF (2009). A combined remote Raman and LIBS instrument for characterizing minerals with 532nm laser excitation. Spectrochim. Acta Part A Mol. Biomol. Spectrosc..

[8] Angel SM, Gomer NR, Sharma SK, McKay C (2011). Remote Raman spectroscopy for planetary exploration: A review. Appl. Spectrosc..

[9] Berlanga G (2019). Remote Raman spectroscopy of natural rocks. Appl. Opt..

[10] Beyssac O (2020). New trends in Raman spectroscopy: From high-resolution geochemistry to planetary exploration. Elements.

[11] Rull F (2022). Spectroscopic study of terrestrial analogues to support rover missions to Mars e A Raman-centred review. Anal. Chim. Acta.

[12] Buseck P, Beyssac O (2014). From organic matter to graphite: Graphitization. Elements.

[13] Rull F (2017). The Raman laser spectrometer for the ExoMars rover mission to Mars. Astrobiology.

[14] Bhartia R (2021). Perseverance’s scanning habitable environments with Raman and luminescence for organics and chemicals (SHERLOC) investigation. Space Sci. Rev..

[15] Maurice S (2021). The SuperCam instrument suite on the Mars 2020 rover: Science objectives and mast-unit description. Space Sci. Rev..

[16] Wiens RC (2021). The SuperCam instrument suite on the NASA Mars 2020 rover: Body unit and combined system tests. Space Sci. Rev..

[17] Hagelschuer, T. *et al.* RAX: The Raman Spectrometer for the MMX Phobos Rover. in *73rd IAC* (2022).

[18] Schröder, S. *et al.* RAX: The Raman Spectrometer on the MMX Rover for in-situ Surface Analysis on Phobos. in *54th LPSC* (2023).

[19] Farley KA (2020). Mars 2020 mission overview. Space Sci. Rev..

[20] Farley KA (2022). Aqueously altered igneous rocks sampled on the floor of Jezero crater, Mars. Science.

[21] Meslin, P.-Y. *et al.* Evidence for perchlorate and sulfate salts in Jezero Crater, Mars from SuperCam observations. in *53rd LPSC* 2 (2022).

[22] Scheller EL (2022). Aqueous alteration processes in Jezero crater, Mars—implications for organic geochemistry. Science.

[23] Wiens RC (2022). Compositionally and density stratified igneous terrain in Jezero crater, Mars. Sci. Adv..

[24] Razzell Hollis J (2022). The power of paired proximity science observations: Co-located data from SHERLOC and PIXL on Mars. Icarus.

[25] Beyssac O (2023). Petrological traverse of the olivine cumulate Séítah formation at Jezero crater, Mars: A perspective from SuperCam onboard perseverance. J. Geophys. Res. Planets.

[26] Clavé E (2023). Carbonate detection with SuperCam in igneous rocks on the floor of Jezero crater, Mars. J. Geophys. Res. Planets.

[27] Corpolongo A (2023). SHERLOC Raman mineral class detections of the Mars 2020 crater floor campaign. J. Geophys. Res. Planets.

[28] Lopez-Reyes, G. *et al.* Anhydrite detection by Raman spectroscopy with SuperCam at the Jezero Deta, Mars. in *54th LPSC* (2023).

[29] Patel MR, Zarnecki JC, Catling DC (2002). Ultraviolet radiation on the surface of Mars and the Beagle 2 UV sensor. Planet. Space Sci..

[30] Dartnell LR, Deshorger L, Ward JM, Coates AJ (2007). Martian sub-surface ionising radiation: Biosignatures and geology. Biogeosciences.

[31] Hassler DM (2014). Mars’ surface radiation environment measured with the Mars science laboratory’s curiosity rover. Science.

[32] Boboyarova, Sh. G., Ibragimov, J. D., Mustafakulov, A. A. & Turdiev, R. T. Influence of radiation influced defects in luminescence of quartz crystals. 10.1023/A:1014587806966 (1999).

[33] Leide, A. J., Lloyd, M. J. & Todd, R. I. Raman spectroscopy of ion irradiated SiC: Chemical defects, strain, annealing, and oxidation. https://ui.adsabs.harvard.edu/link_gateway/2020arXiv200414335L/doi:10.48550/arXiv.2004.14335 (2004).

[34] Shlimak I, Butenko A, Kogan E, Kaveh M (2019). Irradiation-induced broadening of the Raman spectra in monolayer graphene. J. Appl. Phys..

[35] Royer C (2023). Impact of UV radiation on the Raman and infrared spectral signatures of sulfates, phosphates and carbonates: Implications for Mars exploration. Icarus.

[36] Cousin A (2022). SuperCam calibration targets on board the perseverance rover: Fabrication and quantitative characterization. Spectrochim. Acta Part B Atom. Spectrosc..

[37] Fau A (2022). Time-resolved Raman and luminescence spectroscopy of synthetic REE-doped hydroxylapatites and natural apatites. msam.

[38] Fau A (2019). Pulsed laser-induced heating of mineral phases: Implications for laser-induced breakdown spectroscopy combined with Raman spectroscopy. Spectroch. Acta Part B Atom. Spectrosc..

[39] Megevand V (2021). Impact of UV radiation on the Raman signal of cystine: Implications for the detection of S-rich organics on Mars. Astrobiology.

[40] Fau A (2019). Spectroscopies Raman et de Luminescence Résolues en Temps Pour L’exploration de MARS.

[41] Manrique JA (2020). SuperCam calibration targets: Design and development. Space Sci. Rev..

[42] Maurice S (2016). ChemCam activities and discoveries during the nominal mission of the Mars Science Laboratory in Gale crater, Mars. J. Anal. At. Spectrom..

[43] Schröder S (2019). Effects of pulsed laser and plasma interaction on Fe, Ni, Ti, and their oxides for LIBS Raman analysis in extraterrestrial environments. J. Raman Spectrosc..

[44] Alsemgeest J, Pavlov SG, Böttger U, Weber I (2022). Effect of LIBS-induced alteration on subsequent Raman analysis of iron sulfides. ACS Earth Space Chem..

[45] Benner SA, Devine KG, Matveeva LN, Powell DH (2000). The missing organic molecules on Mars. PNAS.

[46] Frantseva K, Mueller M, Loes ten Kate I, van der Tak FFS, Greenstreet S (2018). Delivery of organics to Mars through asteroid and comet impacts. Icarus.

[47] Flynn GJ (1996). The delivery of organic matter from asteroids and comets to the early surface of Mars. Earth Moon Planets.

[48] Poch O (2013). Chemical evolution of organic molecules under Mars-like UV radiation conditions simulated in the laboratory with the “Mars organic molecule irradiation and evolution”(MOMIE) setup. Plan. Space Sci..

[49] Poch O, Kaci S, Stalport F, Szopa C, Coll P (2014). Laboratory insights into the chemical and kinetic evolution of several organic molecules under simulated Mars surface UV radiation conditions. Icarus.

[50] Stalport F, Coll P, Szopa C, Raulin F (2014). Search for organic molecules at the Mars surface: The “Martian Organic Material Irradiation and Evolution”(MOMIE) project. Adv. Space Res..

[51] Poch O (2015). Effect of Nontronite Smectite clay on the chemical evolution of several organic molecules under simulated Martian surface ultraviolet radiation conditions. Astrobiology.

[52] Fornaro T, Steele A, Brucato JR (2018). Catalytic/protective properties of Martian minerals and implications for possible origin of life on Mars. Life.

[53] Fox AC, Eigenbrode JL, Freeman KH (2019). Radiolysis of macromolecular organic material in Mars-relevant mineral matrices. J. Geophys. Res. Planets.

[54] Fornaro T (2020). UV irradiation and near infrared characterization of laboratory Mars soil analog samples. Front. Astron. Space Sci..

[55] Bernard, S. *et al.* Irradiation of organics on Mars: Evolution of the Raman signal of the ertalyte target aboard Perseverance. in *54th LPSC* (2023).

[56] Fries MD (2022). The SHERLOC calibration target on the Mars 2020 perseverance rover: Design, operations, outreach, and future human exploration functions. Space Sci. Rev..

[57] Pidgeon RT, Nasdala L, Todt W (1998). Determination of radiation damage ages on parts of zircon grains by Raman microprobe: implications for annealing history and U-Pb stability. Mineral Mag. A.

[58] Nasdala L, Wenzel M, Andrut M, Wirth R, Blaum P (2001). The nature of radiohaloes in biotite: Experimental studies and modeling. Am. Mineral..

[59] Nasdala L (2006). Alpha particle haloes in chlorite and cordierite. Mineral. Petrol..

[60] Krickl R, Nasdala L, Götze J, Grambole D, Wirth R (2008). Alpha-irradiation effects in SiO_2_. Eur. J. Mineral..

[61] Pabst, S. *et al.* Radiation damage in heavy ion-irradiated carbonate minerals investigated by Raman and infrared spectroscopy. *GSI Scientific Report* (2010).

[62] Leon, M. *et al.* Neutron irradiation effects on the structural properties of KU1, KS-4V and I301 silica glasses. *Proc. RADECS* 1–5 (2013).

[63] Wiens, R. C. & Maurice, S. Mars 2020 SuperCam bundle. *NASA Planetary Data System* (2021).

[64] Legett C (2022). Optical calibration of the SuperCam instrument body unit spectrometers. Appl. Opt..

[65] Montagnac G (2018). Spark plasma sintering preparation of reference targets for field spectroscopy on Mars. J. Raman Spectrosc..

[66] Madariaga JM (2022). Homogeneity assessment of the SuperCam calibration targets onboard rover perseverance. Anal. Chim. Acta.

[67] Forni O (2015). First detection of fluorine on Mars: Implications for Gale Crater’s geochemistry. Geophys. Res. Lett..

[68] Liu Y (2022). An olivine cumulate outcrop on the floor of Jezero crater, Mars. Science.

[69] McCubbin FM, Jones RH (2015). Extraterrestrial apatite: Planetary geochemistry to astrobiology. Elements.

[70] Malarewicz V (2023). Raman spectroscopy investigations of the Martian regolith breccia Northwest Africa 7533: A support to in situ Raman spectroscopy on Mars. J. Raman Spectrosc..

[71] O’Shea DC, Bartlett ML, Young RA (1974). Compositional analysis of apatites with laser-Raman spectroscopy: (OH, F, Cl) apatites. Arch. Oral Biol..

[72] Sundius T (1973). Computer fitting of Voigt profiles to Raman lines. J. Raman Spectrosc..

[73] Newville, M., Stensitzki, T., Allen, D. B. & Ingargiola, A. *LMFIT: Non-Linear Least-Square Minimization and Curve-Fitting for Python*. https://zenodo.org/doi/10.5281/zenodo.598352 (2014).

[74] Eilers, P. H. C. & Boelens, H. F. M. Baseline correction with asymmetric least squares smoothing. *Leiden University Medical Centre report* (2005).

