# Supplementary material for: Radiation-induced alteration of apatite on the surface of Mars: first in situ observations with SuperCam Raman onboard Perseverance
Source: Sci Rep. 2024 May 17;14:11284. doi: 10.1038/s41598-024-61494-5 (PMC11101483; doi:10.1038/s41598-024-61494-5)
Supplement: Supplementary file 1 — Supplementary Information. [file 41598_2024_61494_MOESM1_ESM.pdf]

Supplementary materials in support of the paper:

Radiation-induced alteration of apatite on the surface of Mars: First in situ observations with SuperCam Raman onboard Perseverance

E. Clavé*[1] (elise.clave@dlr.de), O. Beyssac[2], S. Bernard[2], C. Royer[3], G. Lopez-Reyes[4], S. Schröder[1], K. Rammelkamp[1], O. Forni[5], A. Fau[5], A. Cousin[5], J. A. Manrique[4], A. Ollila[6], J. M. Madariaga[7], J. Aramendia[7], S. K. Sharma[8], T. Fornaro[9], S. Maurice[5], R.C. Wiens[3]

1- DLR - Institute of Optical Sensor Systems, Berlin, Germany
2- Institut de Minéralogie, de Physique des Matériaux et de Cosmochimie, CNRS UMR 7590, Muséum National d'Histoire Naturelle, Sorbonne Université, Paris, France
3- Earth, Atmospheric, and Planetary Sciences, Purdue University, West Lafayette, IN, United States
4- Research Group ERICA, Universidad de Valladolid, Valladolid, Spain
5- Institut de Recherche en Astrophysique et Planétologie, Université de Toulouse, CNRS, CNES, Toulouse, France
6- Los Alamos National Laboratory, Los Alamos, NM, United States
7- Dept. Anal. Chem., University of the Basque Country UPV/EHU, 48940 Leioa, Spain
8- Hawaii Inst. of Geophys. & Planetology, Univ. of Hawaii, Honolulu, HI-96822, United States
9- INAF-Astrophysical Observatory of Arcetri, Largo E. Fermi 5, 50125 Firenze, Italy

Sol	Time (ltst)	Focus (mm)	sp p co-adds	N co-adds	Gain	Freq. (Hz)	Description
51	13:58	1565	1	200	3200	10	
55	16:23	1562	1	150	2900	10	Gain test
55	16:24	1562	1	150	3200	10	Gain test
55	16:25	1562	1	150	3400	10	Gain test
60	14:17	1564	1	100	3400	10	co-add test
60	14:17	1564	5	20	3400	10	co-add test
60	14:18	1564	10	10	3400	10	co-add test
60	14 :19	1564	20	5	3400	10	co-add test
149	11:53	1561	1	200	3400	10	co-add test
356	13:42	1563	10	20	3400	10	3Hz test
356	13:44	1563	1	200	3400	10	3Hz test
356	13:48	1563	10	20	3400	3	3Hz test
356	13:52	1563	1	200	3400	3	3Hz test
484	13:05	1562	10	10	3400	10	nominal obs.
606	15:55	1562	10	10	3400	10	nominal obs.
636	12:04	1561	10	20	3400	10	dust charac.
636	12:07	1562	10	20	3400	10	dust charac.
715	15:56	1563	10	10	3400	10	nominal obs.
836	15:31	1562	10	10	3400	10	nominal obs.
888	12:50	1563	10	10	3400	10	nominal obs.
936	10:53	1562	10	10	3400	10	nominal obs.

Table S1 – Summary of the parameters used for the Raman observations of the apatite calibration target with SuperCam Raman over the first 950 sols of the Mars2020 mission: the sol and time of observation (ltst), the position of the focus (i.e. distance to the target), the number of shots accumulated on the CCD for 1 co-add, the number of co-adds acquired, the gain value and repetition rate used. The last column provides a short description of the activity.

We have acquired Raman spectra on the TAPAG target on 13 different sols between sols 51 and 936; the data acquired on sol 352 are the only spectra acquired with lower laser current values (current of diodes of 140.0 A instead of 155.0 A), and are therefore not used in this study. The gain test (sol 55) and co-add test (sol 60) aimed to observe the influence of the gain amplification, and the number of shots accumulated on the CCD for a total of 100 laser shots averaged, respectively. On sol 356, we checked that acquiring the spectra with a laser repetition rate of 3 shots per second gave similar results as the nominal frequency of 10 Hz. Finally, on sol 636, we studied the effect of a thin layer of dust on the surface of the target, by acquiring Raman spectra before and after the dust removal. The dust that had accumulated on the target over ~40 sols (since the previous LIBS analysis on this target) was deliberately moved away as a result of a LIBS measurement which is accompanied by a laser-induced shockwave.

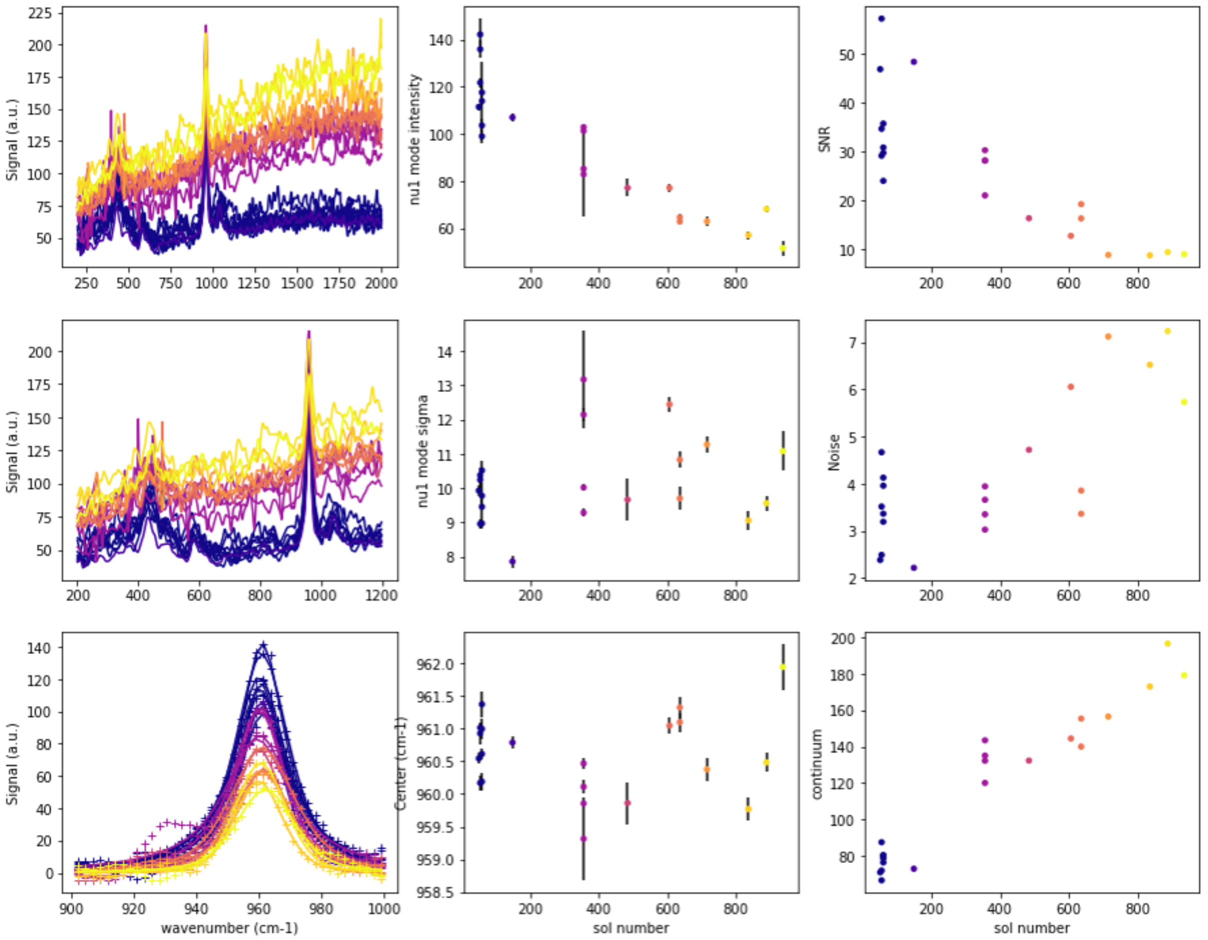

Figure S1 – Mars Raman spectra acquired with SuperCam on apatite SCCT, on different sols throughout the mission (not normalized). The plots are color coded with sol number. First column, spectra (x axis shows wavenumber in cm-1): top, whole spectra; middle, zoom on main Raman region of interest; bottom, close-view in the 900 – 1000 cm^{-1} spectral range; baseline corrected experimental data (+) and fitted Pseudo-Voigt profile (full lines). Other two columns, characteristics of the Raman ν_1 mode (intensity, width, center, SNR), noise and continuum signal evolutions. The error bars represent the uncertainty values of the parameters derived from the peak fitting procedure. All plots in the two right columns share the same x axis.

In the spectra presented here (and all "non-normalized spectra" in this study), we correct the influence of the number of spectra accumulated on the CCD by dividing each spectrum by the number of laser shots accumulated on the CCD per *co-add* (see Table S1). Since the different *co-adds* are averaged, variable *co-add* numbers may result in variable noise levels, but should not affect the signal intensity. However, it is expected to affect the noise level, and hence the signal-to-noise ratio (SNR). The influence of different amplification (gain) values is corrected as part of the IRF correction [1]. The continuum value presented in the lower right plot corresponds to the mean value of the signal over the entire spectral range for each spectrum (the one used to normalize the spectra presented in the paper).

A clear increase of the continuum signal is visible in the spectra, as the time spent on Mars increases; the difference between the first and last spectra, acquired more than one Mars year (669 sols) apart, is of a factor ~2.5. We also observe a clear decrease of the intensity of the ν_1 Raman mode of apatite throughout the mission; the intensity is reduced to about half its initial value over the first Martian year. We observe higher noise levels in the latest spectra, which is expected as part of the noise is correlated to the signal intensity, and the background signal is higher in the latest spectra. However, the correlation with sol number is weak, probably due to the influence of other parameters on the noise level, including the total number of shots used for the different observations (see Table 1 and Figure S1-A). Finally, the width of the peak and its position vary a little (sigma between ~8 and ~13 cm^{-1} and center between

~959.5 and ~961.5 cm^{-1}), but with no visible correlation to the time spent on the surface of Mars and likely not significant. We did not identify a specific acquisition parameter that could explain these small variations.

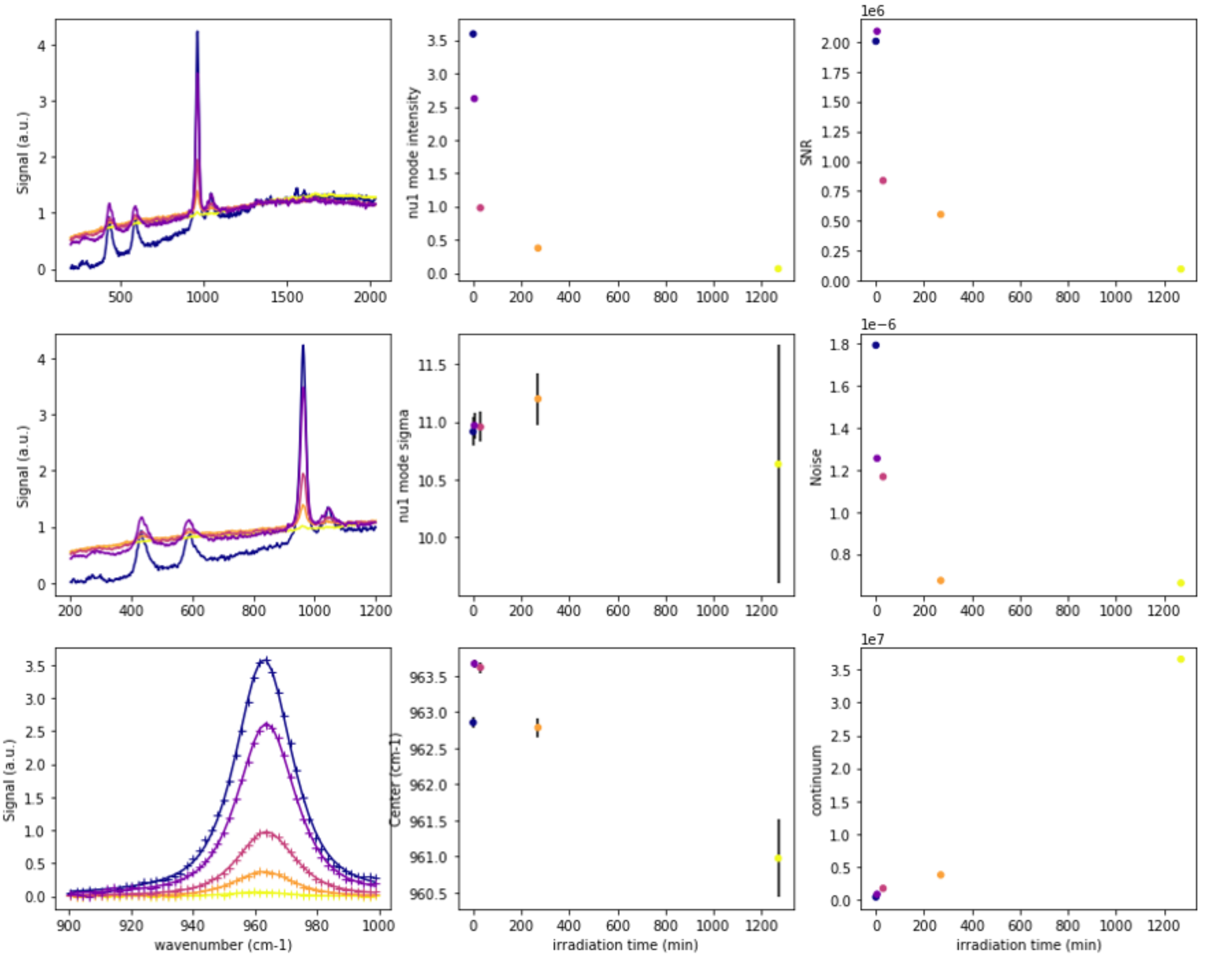

Figure S2 – Laboratory Raman spectra acquired on an apatite target, after different times of UV irradiation (0, 5 min, 30 min, 1270 min, 270 min), normalized to the mean signal. The plots are color coded with UV irradiation time. First column, spectra: top, whole spectra; middle, zoom around mode of interest before fit; bottom, close-view in the 900 – 1000 cm^{-1} spectral range; baseline corrected experimental data (+) and fitted Pseudo-Voigt profile (full lines). Other two columns, characteristics of the Raman ν_1 mode (intensity, width, center, SNR), noise (divided by 10^4) and continuum signal evolutions. The error bars represent the uncertainty values of the parameters derived from the peak fitting procedure.

We also note that the intensity of both Raman modes and continuum signal varies with the time spent under UV irradiation. Overall, we observe a decrease of the Raman signal (minus ~25% in 270 min irradiation), while the background signal increases (almost times 3 in 270 min of UV irradiation). However, the spectra acquired after 5 minutes of irradiation do not quite follow the trend; they show variable Raman signal, and lower background signal than the spectrum acquired before starting irradiation. As expected, the noise level is correlated to the intensity of the continuum signal, and this increases with UV irradiation time overall.

The width of the peak appears mostly stable (within error bars) throughout the series. We observe variability in the position of the ν_1 mode, which we attribute to instability of the setup.

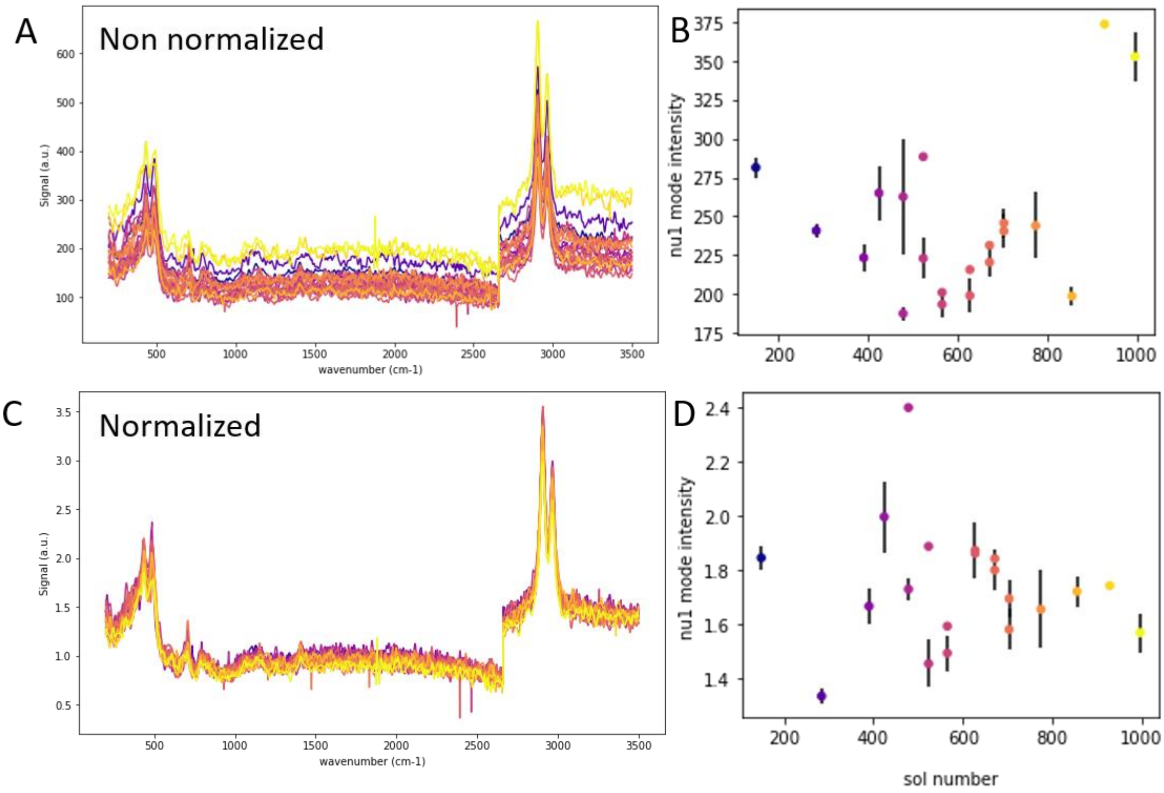

Figure S3 – Raman spectra of the white paint of the calibration target holder, acquired between sols 149 and 928 with SuperCam. (A-B) Data before normalization. (C-D) Data normalized to the mean signal. The mode at 2900 cm^{-1} was fitted with a Pseudo-Voigt profile, to extract the Raman signal as for the apatite target; the corresponding Raman signal intensity are shown in B and D, revealing no particular trend of the Raman signal with time. Despite a certain variability in the signals due to acquisition conditions, this shows that the evolution observed in the Raman spectra of the apatite SCCT are not due to an evolution of the instrument.

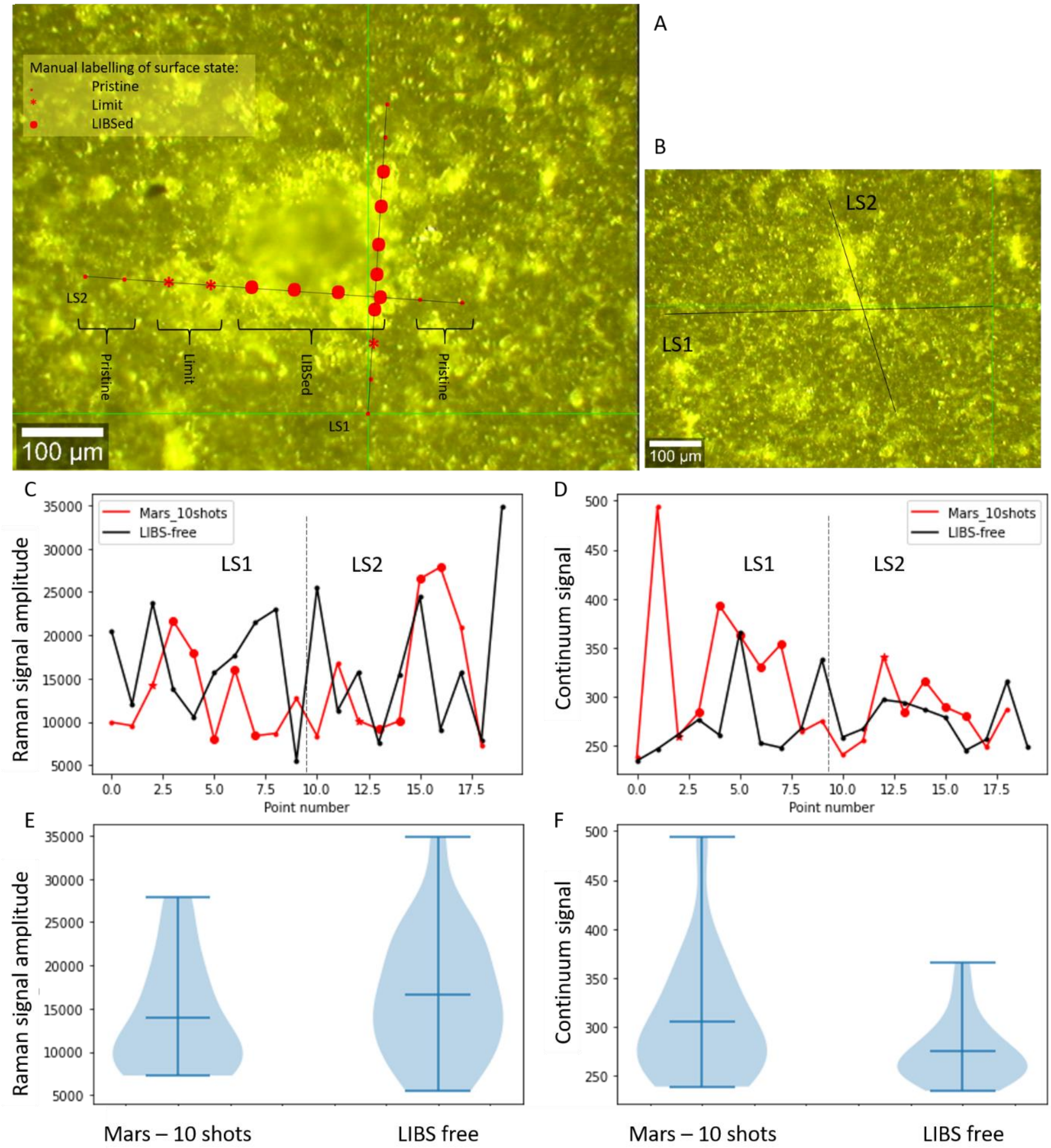

Figure S4 – Comparison of the Raman spectra acquired on pristine versus LIBS-affected areas on the apatite SCCT. LIBS performed in Mars atmospheric conditions in DLR-OS, Berlin, Germany, using 10 laser shots. Micro-Raman spectra acquired at DLR-OS: two 10-points line scans across LIBS crater and two line scans in non-LIBS-affected area (same strategy as in Schröder et al. [2]). Each analysis point was manually labelled as “LIBS-ed”, “pristine” or “limit”, when it was hard to judge. The ν_1 mode of apatite was fitted to extract its intensity; the continuum is extracted by using the average signal above 1500 cm^{-1}. A- Position of the analyzed points, and labelling of surface state for each point. B- Pristine area used for reference, 10 analysis points were distributed regularly along the two indicated line scans; C and D- Raman and continuum intensities, respectively, for the 20 points acquired on each area: across the LIBS crater in red and in the pristine area in black. For the points acquired across the crater, the markers indicate the surface state. E and D- Distribution of the Raman and continuum intensities. We can see there’s no significant difference between the LIBSed and pristine areas.

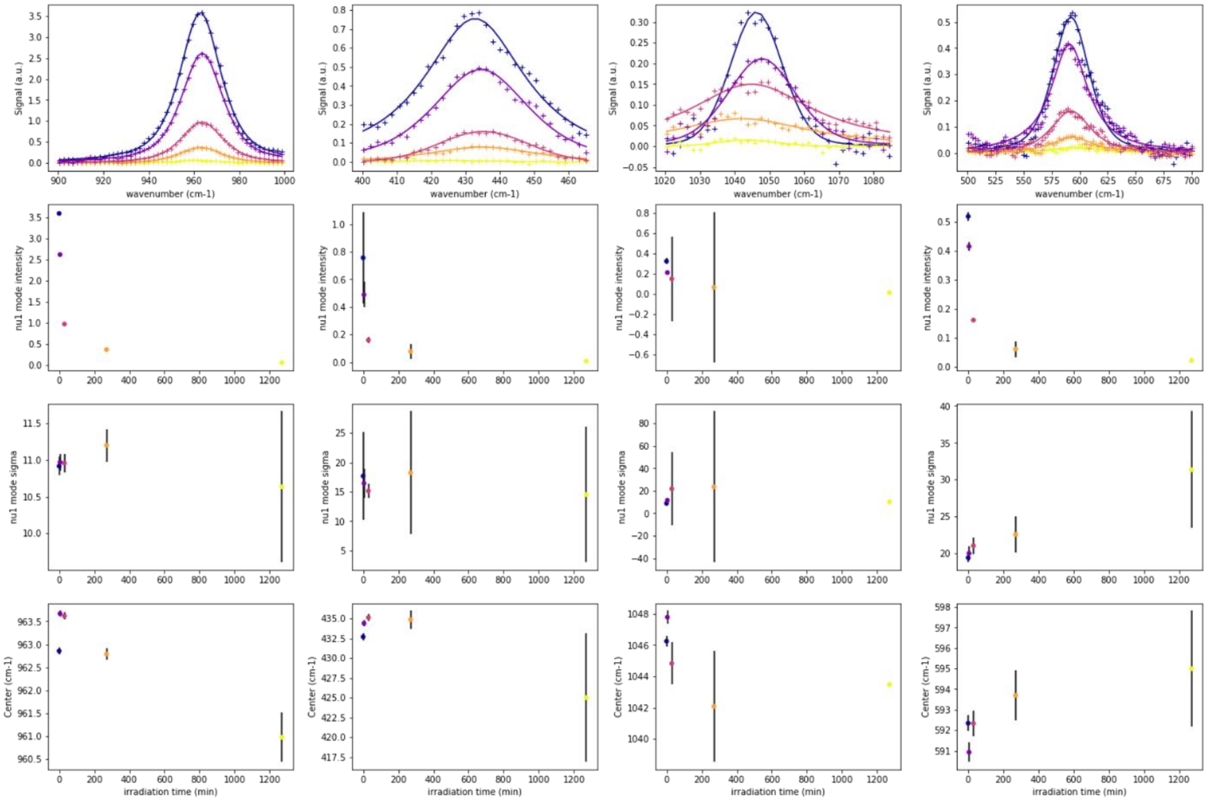

Figure S5 – Comparison of the evolution of the apatite Raman modes, as observed during the UV-irradiation experiment in the laboratory (spectra normalized to mean signal). The four columns correspond to the ν_1, ν_2, ν_3 and ν_4 Raman modes of apatite, respectively. The four lines show the fit of the mode with a Pseudo-Voigt model, the extracted line intensity (area), width (sigma) and position (line center, in cm^{-1}).

References:

1. Wiens, R. C. & Maurice, S. Mars 2020 SuperCam bundle [Dataset]. *NASA Planetary Data System* (2021).

2. Schröder, S. *et al.* Effects of pulsed laser and plasma interaction on Fe, Ni, Ti, and their oxides for LIBS Raman analysis in extraterrestrial environments. *Journal of Raman Spectroscopy* (2019).
